# Liquid biopsies reveal dual compartments of cancer risk from tumor and host-derived mutations

**DOI:** 10.1093/bioinformatics/btag491

**Published:** 2026-08-21

**Authors:** Federica Malighetti, Ivan Civettini, Andrea Aroldi, Alberto Maria Villa, Matteo Villa, Luca Sala, Nicoletta Cordani, Massimiliano Cadamuro, Diego Cortinovis, Rocco Piazza, Luca Mologni, Daniele Ramazzotti

**Affiliations:** Department of Medicine and Surgery, University of Milano-Bicocca, Milano, Italy; Department of Medicine and Surgery, University of Milano-Bicocca, Milano, Italy; Experimental Immunology Unit, IRCCS San Raffaele Scientific Institute, Milan, Italy; Department of Medicine and Surgery, University of Milano-Bicocca, Milano, Italy; Fondazione IRCCS San Gerardo dei Tintori di Monza, Monza, Italy; Department of Medicine and Surgery, University of Milano-Bicocca, Milano, Italy; Department of Medicine and Surgery, University of Milano-Bicocca, Milano, Italy; Fondazione IRCCS San Gerardo dei Tintori di Monza, Monza, Italy; Department of Medicine and Surgery, University of Milano-Bicocca, Milano, Italy; Department of Medicine and Surgery, University of Milano-Bicocca, Milano, Italy; Fondazione IRCCS San Gerardo dei Tintori di Monza, Monza, Italy; Department of Medicine and Surgery, University of Milano-Bicocca, Milano, Italy; Fondazione IRCCS San Gerardo dei Tintori di Monza, Monza, Italy; Department of Medicine and Surgery, University of Milano-Bicocca, Milano, Italy; Fondazione IRCCS San Gerardo dei Tintori di Monza, Monza, Italy; Department of Medicine and Surgery, University of Milano-Bicocca, Milano, Italy; Fondazione IRCCS San Gerardo dei Tintori di Monza, Monza, Italy; Department of Medicine and Surgery, University of Milano-Bicocca, Milano, Italy; Fondazione IRCCS San Gerardo dei Tintori di Monza, Monza, Italy

## Abstract

**Motivation:**

Circulating tumor DNA (ctDNA) and clonal hematopoiesis of indeterminate potential (CHIP) are two biologically distinct sources of somatic mutations detectable in blood. While ctDNA captures tumor-intrinsic alterations, CHIP arises from age-related hematopoietic clones and is often considered background noise. Here, we conduct a large-scale, tumor-type–resolved analysis of over 9000 patients with CHIP data and 1500 patients with ctDNA data across solid tumors profiled at Memorial Sloan Kettering Cancer Center.

**Results:**

Our results reveal that CHIP and ctDNA mutations exhibit non-overlapping, clinically meaningful signals. CHIP mutations, particularly in DNA damage response and epigenetic regulators (e.g. PPM1D, CHEK2, ATM, TP53, ASXL1), are associated with worse overall survival, increased metastatic potential, and site-specific dissemination. ctDNA mutations in canonical oncogenic drivers (e.g. TP53, EGFR, KRAS, STK11) reflect tumor aggressiveness and correlate with poor prognosis and metastasis across multiple cancer types. Joint modeling in lung adenocarcinoma confirms the independent prognostic contributions of both compartments. Additionally, longitudinal clonal analysis links specific CHIP mutations to the emergence of hematologic malignancies under therapeutic pressure. These findings support a dual-compartment model of liquid biopsy, in which tumor- and host-derived mutations jointly inform on cancer risk, progression, and metastatic behavior. Integrating both compartments may enhance the clinical utility of blood-based biomarkers in oncology.

**Availability:**

All genomic and clinical data used in this study are available through cBioPortal. Summarized outputs and processed results tables are provided in Supplementary Data.

## 1 Introduction

Liquid biopsy technologies are revolutionizing cancer diagnosis and management by enabling the detection of tumor-associated genomic alterations through blood-based sequencing. Unlike traditional tissue biopsies, which are invasive and limited to a single tumor site, liquid biopsies provide a minimally invasive and dynamic snapshot of cancer biology, capturing both tumor evolution and systemic host responses. They allow for the detection of minimal residual disease, real-time tracking of tumor evolution, assessment of tumor heterogeneity, and monitoring of host interactions. With their ease of use and expanding clinical applications, liquid biopsies are rapidly becoming an indispensable tool in precision oncology, empowering oncologists to make more informed, patient-centered decisions. Among the most established applications are the analysis of circulating tumor DNA (ctDNA) and the profiling of somatic mutations in peripheral blood cells, especially those linked to clonal hematopoiesis of indeterminate potential (CHIP) (Reed *et al.* 2023). CHIP refers to the age-associated accumulation of somatic mutations in hematopoietic stem and progenitor cells, which leads to the clonal expansion of mutated blood cells in the absence of overt hematologic disease (Xie *et al.* 2014). While originally studied in relation to cardiovascular and hematologic conditions, CHIP has recently emerged as a prognostic factor in patients with solid tumors (Stein *et al.* 2022, Buttigieg and Rauh 2023). Importantly, recent evidence shows that CHIP mutations can infiltrate tumor tissues, a phenomenon termed tumor-infiltrating clonal hematopoiesis (TI-CH) (Pich *et al.* 2025). This indicates that CHIP clones are not merely present in the bloodstream. They may exert non-cell-autonomous influences on tumor progression, such as through immune modulation, systemic inflammation, or effects on the metastatic niche. In parallel, ctDNA analysis has become a powerful tool to non-invasively profile tumor-specific mutations, enabling real-time monitoring of tumor burden, therapeutic resistance, and clonal selection (Dalle Fratte *et al.* 2020, Villa *et al.* 2024). Although both CHIP and ctDNA mutations are detectable in the same blood sample, they arise from distinct biological compartments, host and tumor, respectively and reflect fundamentally different processes: one related to host aging and immune status, the other to tumor-intrinsic genomic dynamics. Despite their co-occurrence, studies often have treated CHIP as background signal to be excluded from ctDNA analysis. Whether CHIP and ctDNA mutations provide redundant, additive, or orthogonal prognostic information remains unclear (Ococks *et al.* 2021). Moreover, their joint contribution to key clinical endpoints such as patient survival, metastatic spread, or organ-specific dissemination, has not been systematically studied in large, well-annotated patient cohorts. Here, we present an integrative, tumor-type–resolved analysis of somatic mutations derived from CHIP and ctDNA across more than 9000 patients with CHIP profiles and over 1500 with ctDNA data, profiled at Memorial Sloan Kettering Cancer Center (MSKCC) (Nguyen *et al.* 2022, Stonestrom *et al.* 2024, Jee, Brannon, *et al.* 2024, Jee, Fong, *et al.* 2024). We investigate the prognostic and metastatic impact of these mutations across solid tumor types, incorporating multivariable models adjusted for age, gender, and tumor context. In addition to demonstrating the non-redundant clinical signals from the two compartments, we explore their joint predictive value, their relationship with site-specific metastatic tropism, and the clonal trajectories of CHIP mutations in hematologic cancer transformation. Our findings support a dual-compartment model of liquid biopsy, wherein both tumor- and host-derived signals contribute complementary insights into cancer progression and may improve risk stratification in precision oncology.

## 2 Methods

### 2.1 Patient cohorts and genomic data

All data analyzed in this study were obtained from publicly available datasets hosted on cBioPortal, originally generated by the Memorial Sloan Kettering Cancer Center (MSKCC). Genomic profiling was conducted using the MSK-IMPACT, MSK-ACCESS and MSK-HEME platforms, next-generation sequencing assays targeting several hundred cancer-associated genes in matched tumor tissue, peripheral blood mononuclear cells (PBMCs), and plasma.

To generate the cohort for the CHIP analyses, we intersected data from three studies: the first two comprising 35 884 patients with solid tumors (Nguyen *et al.* 2022, Jee, Fong, *et al.* 2024) and another including 12 983 patients who underwent somatic mutation profiling of PBMCs (Stonestrom *et al.* 2024). This intersection resulted in 9161 patients with both tumor and CHIP sequencing data. Only patients with complete clinical annotation were considered. Analyses were restricted to tumor types with at least 50 evaluable patients, resulting in 24 cancer types included in CHIP-based models. The ctDNA cohort consisted of 3348 patients profiled via plasma-based tumor genotyping (Jee, Brannon, *et al.* 2024). ctDNA mutations reflect alterations detected at the time of plasma-based sequencing and may therefore capture both baseline tumor biology and progression- or therapy-associated events. After matching with solid tumor data (Nguyen *et al.* 2022, Jee, Fong, *et al.* 2024), 1534 patients with both ctDNA and clinical data were retained. 5 tumor types met the ≥50-patient threshold and were included in ctDNA-based analyses. Clinical and mutational data for hematologic malignancies (Ptashkin *et al.* 2023) were considered for 1937 patients, of which 32 also had CHIP data (Stonestrom *et al.* 2024).

### 2.2 Mutation curation

For all datasets, we considered only somatic, non-synonymous mutations for downstream analysis as the source datasets were generated using targeted cancer sequencing panels optimized for coding cancer-associated genes. In this context, synonymous and non-coding variants are generally less informative and less consistently covered across platforms. For CHIP, we retained mutations identified in PBMCs, following established definitions. For ctDNA, mutations were considered tumor-derived based on annotations provided in the MSK bioinformatics pipeline. To ensure adequate power and model stability, we restricted statistical analyses to genes mutated in at least 5 patients per tumor type, while genes mutated in fewer patients were excluded from the multivariable models. Gender (binary) and age (continuous) were included as covariates in all models, given the established association between aging and CHIP prevalence.

### 2.3 Clinical endpoints

Three clinical endpoints were evaluated:

- Overall survival (OS): defined as the time from cancer diagnosis to death from any cause, with right-censoring at five years.
- Metastatic status: a binary variable (yes/no), indicating the presence of distant metastasis at the time of sequencing.
- Metastatic tropism: the anatomical distribution of metastases (e.g. lung, liver, bone), assessed in exploratory analyses and limited to patients with detailed metastatic site annotation.

### 2.4 Statistical analysis

All statistical analyses were performed in R (version 4.3.0). For each tumor type and gene, separate multivariable models were fitted for CHIP and ctDNA-derived mutations.

- Cox proportional hazards models (via the survival package) were used to assess associations with overall survival.
- Logistic regression models (glm, family = binomial) were used to evaluate associations with binary metastatic status.
- Multinomial logistic regression (vglm function, VGAM package) was used to model associations with metastatic tropism. Here, the metastatic site served as the categorical outcome, and the primary tumor location was used as the reference category.

Each gene was modeled as a binary variable (mutated vs. wild-type). Only genes mutated in ≥5 patients within a tumor type were retained to avoid model overfitting and reduce instability of estimates in low-frequency subgroups, with results from these small groups considered exploratory and hypothesis-generating. Associations were interpreted based on nominal significance (*P* < 0.05) and were prioritized for interpretation based on biological plausibility, effect size, and consistency across tumor types. Results are reported as hazard ratios (HR) or odds ratios (OR) respectively for Cox and logistic analyses.

In a subset of tumor types with at least 50 patients harboring both CHIP and ctDNA data, we performed combined multivariable models including age, gender, CHIP mutations, and ctDNA mutations as covariates. The same statistical criteria and modeling approach were applied. Due to the limited number of patients with overlapping CHIP and ctDNA data within the same tumor type, stable and interpretable joint models could only be obtained for lung adenocarcinoma.

## 3 Results

### 3.1 Integrated analysis of liquid biopsy-derived mutations in solid tumors

Liquid biopsy-based genomic profiling is transforming cancer care by enabling minimally invasive characterization of tumor biology and host factors. Two key approaches, clonal hematopoiesis of indeterminate potential (CHIP) analysis and circulating tumor DNA (ctDNA) sequencing, can offer complementary insights: CHIP reflects somatic evolution of the hematopoietic system, while ctDNA captures tumor-derived alterations released into the bloodstream. However, their relative prognostic and predictive value across diverse solid tumors remains poorly understood.

To systematically investigate these two liquid biopsy compartments, we leveraged large-scale genomic and clinical datasets from patients with solid tumors (see Methods). We considered a reference population of 35 884 patients with solid cancers profiled by targeted sequencing platforms (Nguyen *et al.* 2022, Jee, Fong, *et al.* 2024). We then analyzed a second cohort of 12 983 patients who had undergone peripheral blood sequencing and showed CHIP mutations (Stonestrom *et al.* 2024).

By intersecting these datasets, we identified 9161 patients with both tumor and CHIP mutational profiles. Among these, we further selected individuals with complete clinical annotation specifically, available data on age and gender and restricted our analyses to tumor types with at least 50 evaluable patients.

In parallel, we analyzed a ctDNA dataset comprising 3348 patients who had undergone plasma-based tumor genotyping with available complete data^12^. Intersecting these patients with the previously described cohort of solid tumors patients, we obtained a total of 1534 patients with matched ctDNA and clinical data. Of these, we selected cancer types with at least 50 evaluable patients.

**Figure 1:**
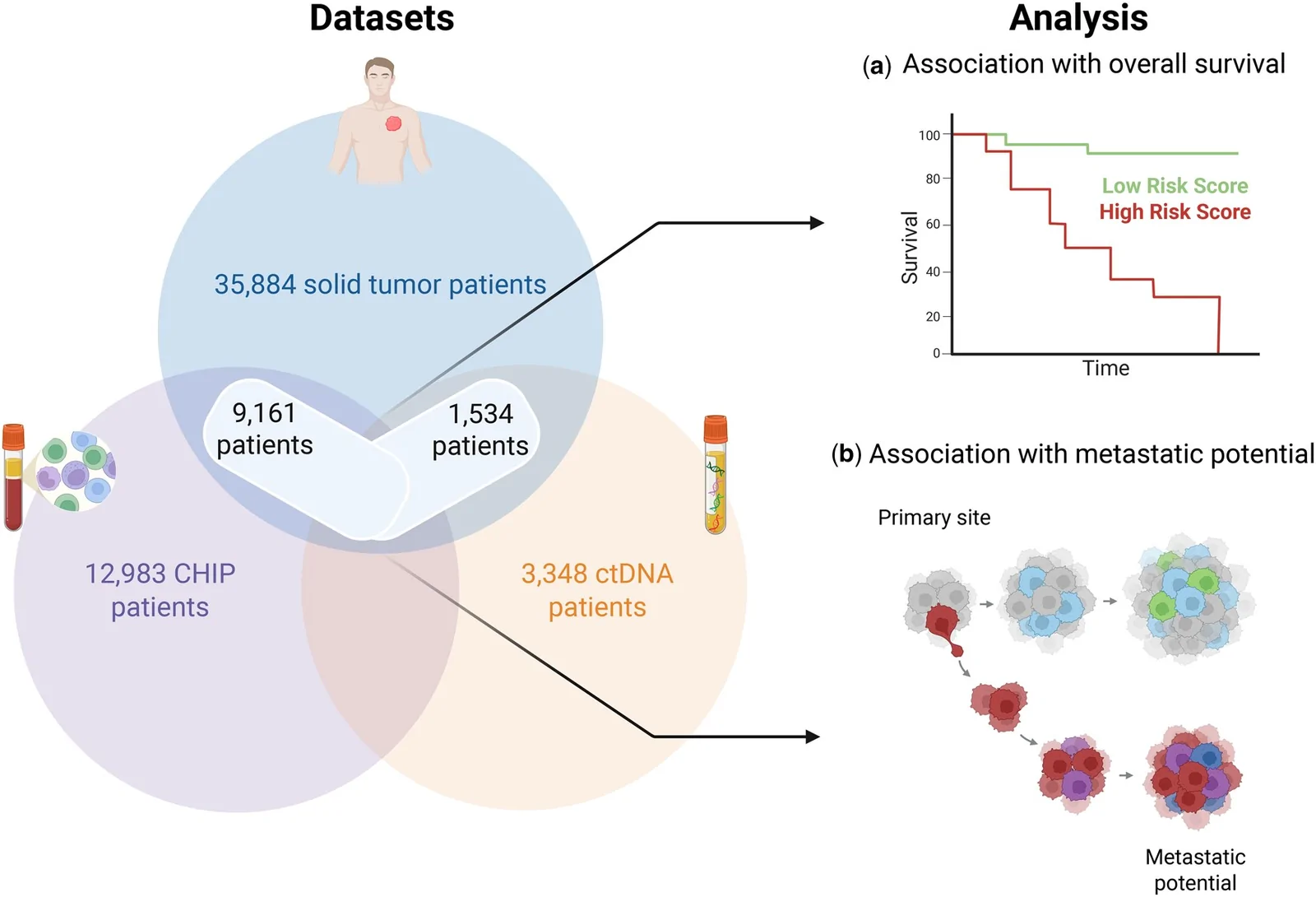
Overview of the datasets analyzed in this study. The solid tumor cohort included 35 884 patients across multiple tumor types (only those with ≥50 patients were analyzed). Among these, 9161 had matched CHIP data and 1534 had ctDNA data, with partial overlap illustrated by the Venn diagram. Downstream analyses focused on patients with solid tumors and either CHIP, ctDNA, or both, evaluating the prognostic and metastatic impact of mutations, including organ-specific metastatic tropism. Figure created with BioRender.com.

For both CHIP and ctDNA cohorts, we conducted two core analyses (Fig. 1):

1. Association with overall survival, modeled using multivariable Cox proportional hazards regression with right-censoring at 5 years.
2. Association with metastatic potential, assessed using multivariable logistic regression with metastasis status as the binary outcome.

All models were adjusted for age and gender and stratified by cancer type to preserve biological interpretability. This study design allows for a systematic, compartment-resolved evaluation of how somatic mutations detected in blood, whether hematopoietic or tumor-derived, relate to patient prognosis and metastatic risk. A summary of cohort sizes and tumor type distribution is provided in Table S1, available as supplementary data at *Bioinformatics* online.

This framework enables a comparative interrogation of host-derived versus tumor-derived circulating mutations and their contributions to cancer aggressiveness across multiple dimensions.

### 3.2 Prognostic value of CHIP mutations across tumor types

**Figure 2:**
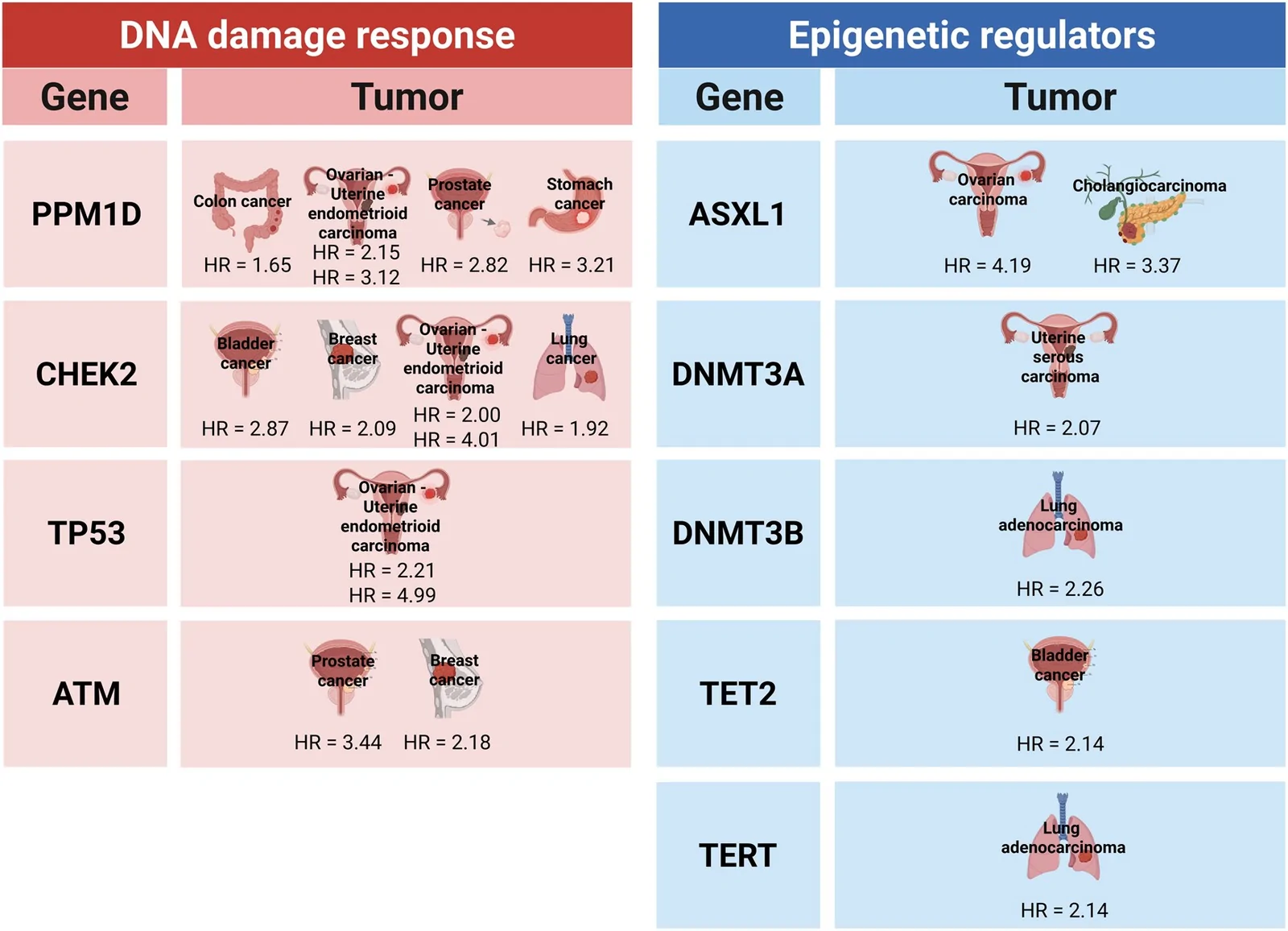
Prognostic associations of CHIP mutations across solid tumor types. The table summarizes genes whose mutations were significantly associated with adverse prognosis, quantified with hazard ratios (HRs), in specific cancers. Genes are grouped into two major functional categories: DNA damage response (DDR, shown in red) and epigenetic regulators (shown in blue). Figure created with BioRender.com.

We identified tumor-type specific prognostic associations between CHIP mutations and patient outcomes. Several recurrent patterns emerged across the evaluated cancer types. These associations were particularly enriched in genes involved in DNA damage response (DDR), such as PPM1D, CHEK2, ATM and TP53 suggesting biologically coherent mechanisms through which hematopoietic clones may influence cancer progression (Fig. 2; Table S2, available as supplementary data at *Bioinformatics* online). PPM1D was the most consistently associated gene, showing adverse prognostic significance in colon (HR = 1.65, *P* = 0.024, *n* = 40), ovary (HR = 2.15, *P* < 0.001, *n* = 95), prostate (HR = 2.82, *P* < 0.001, *n* = 34), stomach (HR = 3.21, *P* = 0.022, *n* = 6), and uterine endometrioid cancer (HR = 3.12, *P* = 0.019, *n* = 23). These findings suggest PPM1D-mediated p53 inhibition as a potential systemic modulator of disease progression across diverse tumor contexts. Similarly, CHEK2 mutations were associated with shorter survival in bladder (HR = 2.87, *P* = 0.024, *n* = 10), breast (HR = 2.09, *P* = 0.040, *n* = 12), ovary (HR = 2.00, *P* = 0.015, *n* = 25), lung (HR = 1.92, *P* = 0.004, *n* = 27), and uterine endometrioid (HR = 4.01, *P* = 0.034, *n* = 6) tumors. Notably, in bladder cancer, ATM mutations also conferred poor prognosis (HR = 3.44, *P* = 0.022, *n* = 7), a signal mirrored in breast cancer (HR = 2.18, *P* = 0.027, *n* = 13), further emphasizing the role of DDR dysregulation via CHIP. Moreover, CHIP-associated TP53 mutations were linked to worse outcomes in ovary (HR = 2.21, *P* = 0.004, *n* = 25) and uterine endometrioid cancer (HR = 4.99, *P* = 0.028, *n* = 7), suggesting potential systemic contributions to tumor aggressiveness. Epigenetic regulators were also associated to worse prognosis. For instance, ASXL1 CHIP mutations were associated with reduced survival in ovary (HR = 4.19, *P* = 0.001, *n* = 9) and cholangiocarcinoma (HR = 3.37, *P* = 0.009, *n* = 6), while DNMT3A had significant effects in uterine serous carcinoma (HR = 2.07, *P* = 0.025, *n* = 38) and DNMT3B lung adenocarcinoma (HR = 2.26, *P* = 0.017, *n* = 12). Additional associations included TET2 mutations in bladder cancer (HR = 2.14, *P* = 0.028, *n* = 28) and TERT mutations in lung adenocarcinoma (HR = 2.14, *P* = 0.023, *n* = 11), highlighting the potential contribution of epigenetic and telomere-related pathways to systemic modulation of cancer outcomes. Together, these results demonstrate that CHIP mutations are clinically relevant in specific solid tumor contexts, particularly those characterized by high genomic instability or immune interaction. The prominence of DDR and epigenetic regulators genes among prognostic markers highlights possible mechanisms through which hematopoietic clones might influence tumor evolution, systemic inflammation, or therapeutic resistance (Fig. 2; Table S2, available as supplementary data at *Bioinformatics* online).

### 3.3 CHIP mutations are associated with metastatic potential in solid tumors

**Figure 3:**
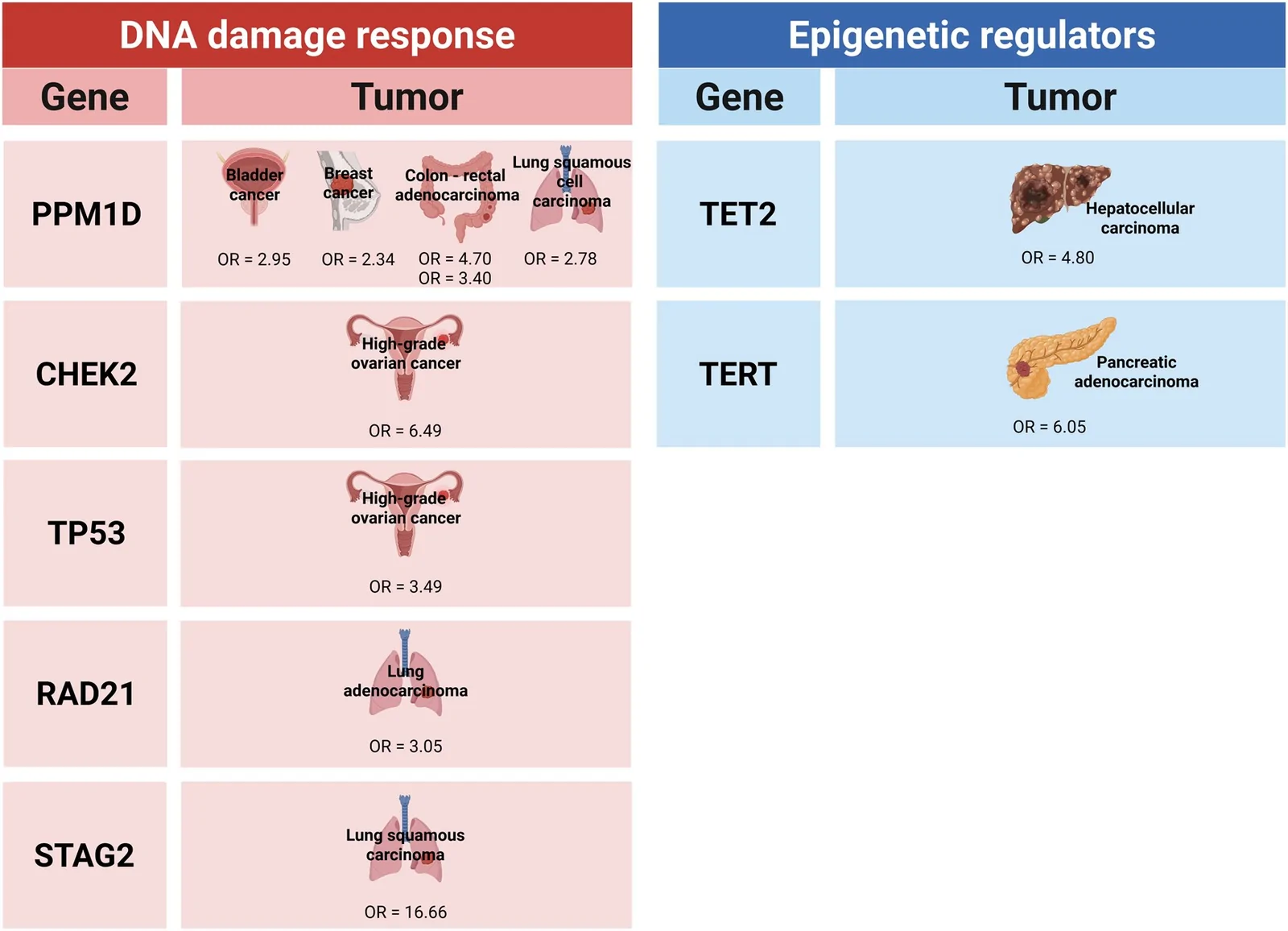
Metastatic associations of CHIP mutations across solid tumor types. The table summarizes genes whose mutations were significantly associated with increased metastatic potential, quantified with odds ratios (ORs), in specific cancers. Genes are grouped into two major functional categories: DNA damage response (DDR, shown in red) and epigenetic regulators (shown in blue). Figure created with BioRender.com.

Several CHIP mutations were significantly associated with increased odds of presenting with metastatic disease, suggesting a role for hematopoietic somatic mutations in modulating tumor-host interactions, systemic inflammation, or immune surveillance escape (Fig. 3; Table S3, available as supplementary data at *Bioinformatics* online).

Among the most recurrent findings, PPM1D mutations emerged as robust predictors of metastatic potential across multiple tumor types, including bladder (OR = 2.95, *P* = 0.005, *n* = 38), breast (OR = 2.34, *P* = 0.015, *n* = 40), colon (OR = 4.70, *P* < 0.001, *n* = 48), lung squamous cell carcinoma (OR = 2.78, *P* = 0.020, *n* = 42), and rectal adenocarcinoma (OR = 3.40, *P* = 0.044, *n* = 14). These results reinforce the notion that PPM1D-driven dysregulation of the DNA damage response may promote a systemic milieu conducive to metastasis.

Additional associations involved canonical DDR genes, such as CHEK2 and TP53, both of which were linked to increased metastatic risk in high-grade ovarian cancer (CHEK2 OR = 6.49, *P* = 0.015, *n* = 26; TP53 OR = 3.49, *P* = 0.033, *n* = 28). Moreover, TET2 mutations were associated with metastasis in hepatocellular carcinoma (OR = 4.80, *P* = 0.049, *n* = 17), suggesting broader involvement of epigenetic regulation in shaping metastatic behavior.

A few less frequent but striking associations included RAD21 in lung adenocarcinoma (OR = 3.05, *P* = 0.037, *n* = 16), STAG2 in lung squamous carcinoma (OR = 16.66, *P* = 0.030, *n* = 6), and TERT in pancreatic adenocarcinoma (OR = 6.05, *P* = 0.041, *n* = 7). These genes, involved in chromosomal cohesion and telomere maintenance, may represent novel systemic mediators of metastatic predisposition.

Altogether, these findings demonstrate that CHIP mutations are not only prognostic but may also predict metastatic phenotypes. The recurrence of DDR and epigenetic genes across multiple cancer types supports the hypothesis that clonal hematopoiesis may influence the systemic microenvironment in ways that promote cancer dissemination (Fig. 3; Table S3, available as supplementary data at *Bioinformatics* online).

### 3.4 ctDNA mutations highlight tumor-intrinsic risk of mortality and metastasis

**Figure 4:**
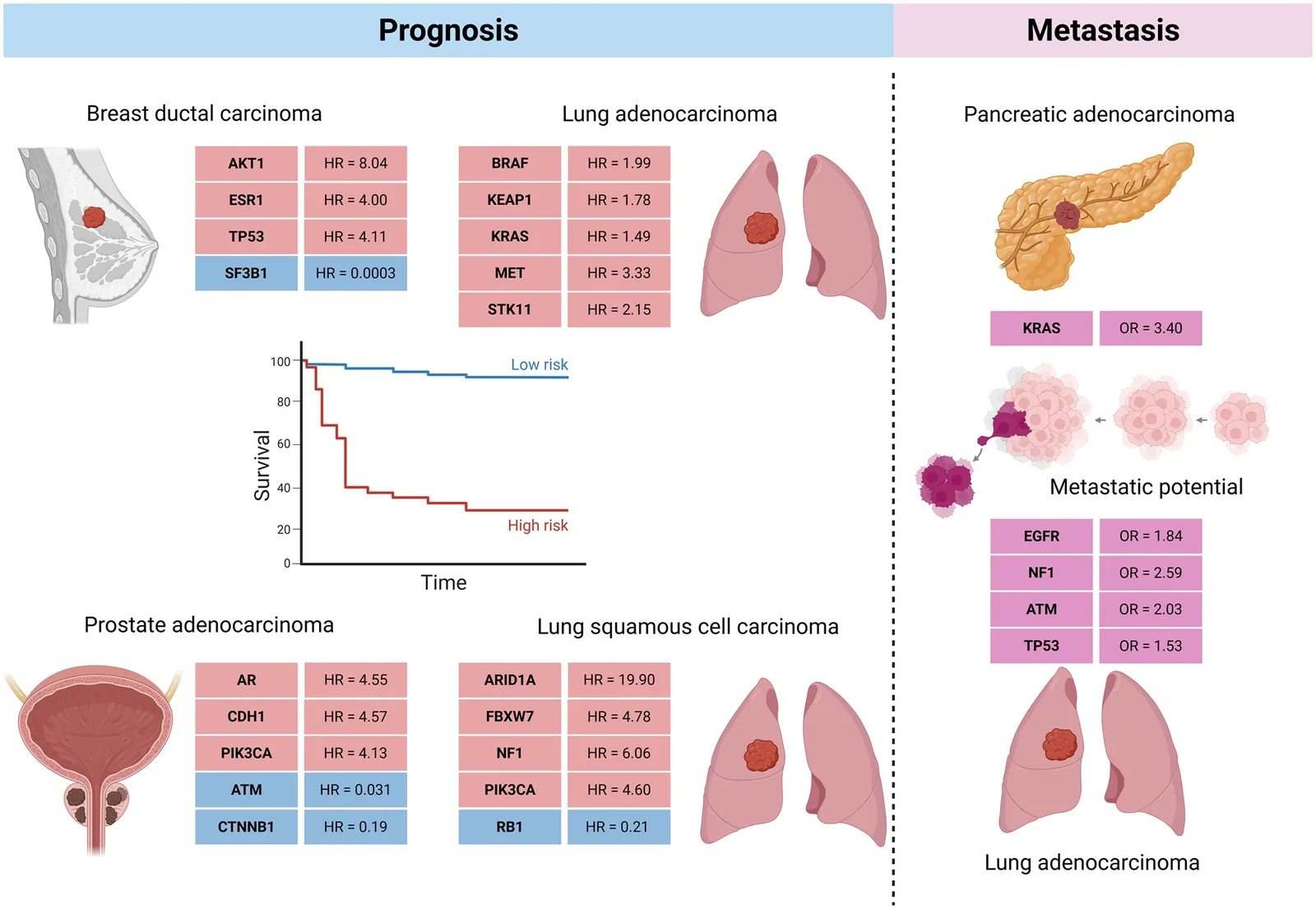
Prognostic and metastatic associations of ctDNA mutations. The left panel shows ctDNA mutations identified in breast ductal carcinoma, lung adenocarcinoma, prostate adenocarcinoma, and lung squamous carcinoma, with unfavorable prognosis highlighted in red and favorable prognosis in blue. The right panel illustrates the association of KRAS mutations in ctDNA from pancreatic adenocarcinoma patients with increased metastatic potential. Figure created with BioRender.com.

We next evaluated the prognostic and metastatic relevance of tumor-derived circulating DNA (ctDNA) mutations in solid tumors (Fig. 4; Tables S4–S5, available as supplementary data at *Bioinformatics* online), applying the same multivariable regression framework used for CHIP to ctDNA data.

In breast ductal carcinoma, mutations in AKT1 (HR = 8.04, *P* = 0.0137, *n* = 8), ESR1 (HR = 4.00, *P* = 0.0134, *n* = 24), and TP53 (HR = 4.11, *P* = 0.0126, *n* = 57) were linked to significantly reduced survival, highlighting the prognostic importance of endocrine resistance and genomic instability. Conversely, SF3B1 mutations were associated with improved outcomes (HR = 0.0003, *P* = 0.0150, *n* = 8), suggesting the presence of a less aggressive molecular subtype.

In lung adenocarcinoma, mutations in BRAF (HR = 1.99, *P* = 0.0371, *n* = 19), KEAP1 (HR = 1.78, *P* = 0.0148, *n* = 56), KRAS (HR = 1.49, *P* = 0.0451, *n* = 114), MET (HR = 3.33, *P* = 0.0011, *n* = 10), and STK11 (HR = 2.15, *P* = 0.0041, *n* = 42) were significantly associated with poor prognosis, implicating MAPK signaling, oxidative stress response, and tumor invasiveness in ctDNA-associated prognosis. Furthermore, this tumor type also exhibited strong associations with metastatic presentation. Notably, EGFR mutations (OR = 1.84, *P* = 0.0074, *n* = 238), NF1 (OR = 2.59, *P* = 0.0386, *n* = 36), ATM (OR = 2.03, *P* = 0.0464, *n* = 49), and TP53 (OR = 1.53, *P* = 0.0273, *n* = 322) all conferred elevated risk, reflecting the contribution of both canonical oncogenic drivers and tumor suppressors in systemic spread.

In lung squamous cell carcinoma, prognostically relevant mutations included ARID1A (HR = 19.90, *P* = 0.0050, *n* = 5), FBXW7 (HR = 4.78, *P* = 0.0475, *n* = 6), NF1 (HR = 6.06, *P* = 0.0357, *n* = 5), and PIK3CA (HR = 4.60, *P* = 0.0283, *n* = 7), supporting a role for epigenetic dysregulation and PI3K pathway activation in aggressive disease. RB1 mutations were associated with better survival (HR = 0.21, *P* = 0.0472, *n* = 7), possibly reflecting a distinct clinical phenotype. Moreover, in prostate adenocarcinoma, ctDNA mutations in AR (HR = 4.55, *P* = 0.0006, *n* = 14), CDH1 (HR = 4.57, *P* = 0.0098, *n* = 5), and PIK3CA (HR = 4.13, *P* = 0.0102, *n* = 6) were associated with reduced survival, while mutations in ATM (HR = 0.031, *P* < 0.0001, *n* = 6) and CTNNB1 (HR = 0.19, *P* = 0.0253, *n* = 4) predicted improved prognosis, underscoring complex interactions between DNA repair, androgen signaling, and cell adhesion.

Finally, in pancreatic adenocarcinoma, KRAS mutations were strongly associated with the metastatic phenotype (OR = 3.40, *P* = 0.0169, *n* = 107), highlighting the central role of RAS pathway activation in early dissemination in this cancer type.

Together, these findings demonstrate that ctDNA mutations encode biologically meaningful and clinically relevant indicators of both survival and metastatic potential (Fig. 4; Tables S4–S5, available as supplementary data at *Bioinformatics* online). The recurrence of pathways such as PI3K/AKT, RAS/MAPK, DNA damage response, and chromatin remodeling suggests that liquid biopsy data may capture convergent mechanisms of cancer progression across anatomically diverse tumor types.

### 3.5 CHIP and ctDNA mutations are associated with site-specific metastatic tropism

**Figure 5:**
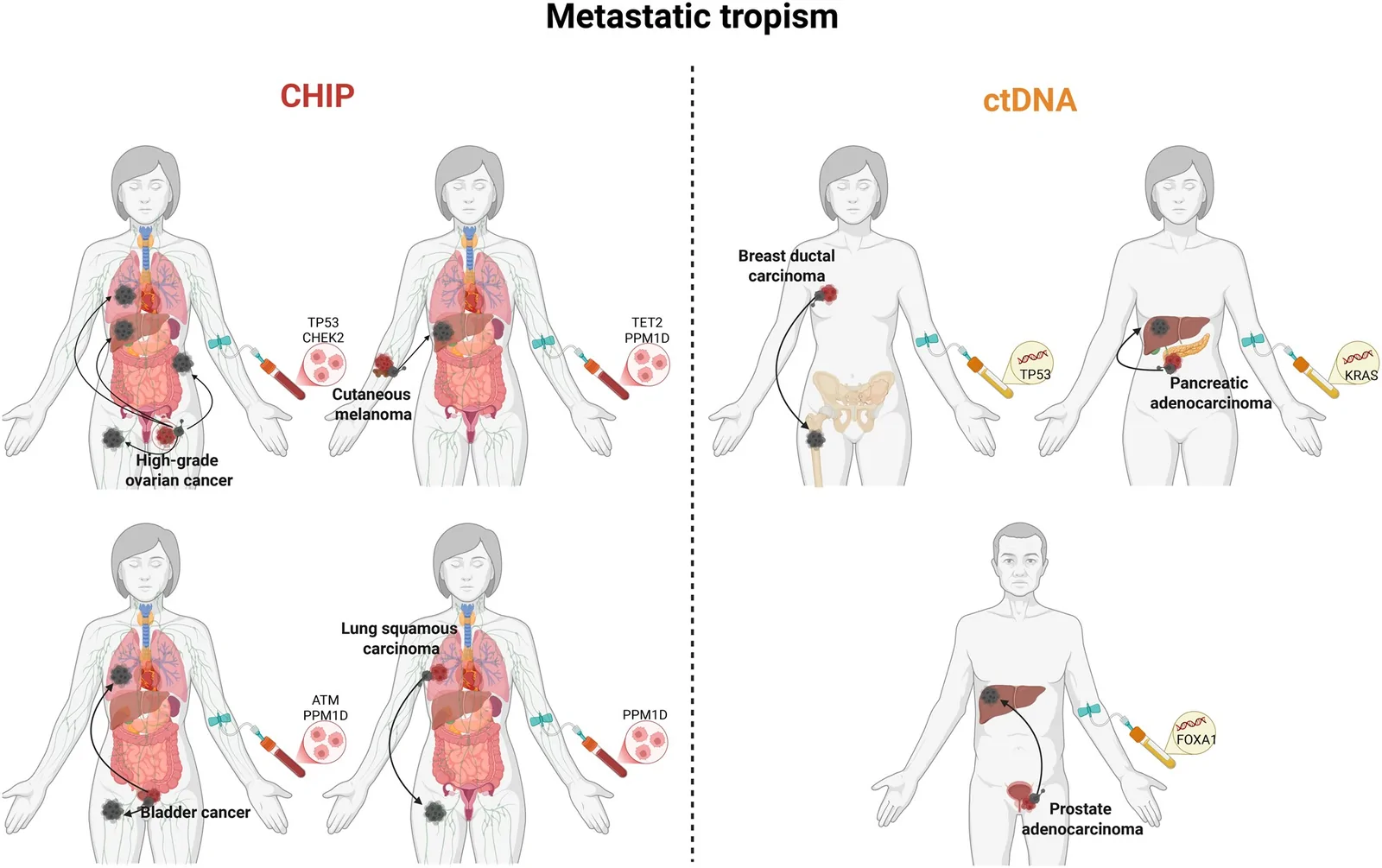
Organ-specific metastatic tropisms associated with mutations detected in CHIP (left) and ctDNA (right) across cancer types. Among CHIP-derived alterations, mutations in DNA damage response and epigenetic regulator genes displayed strong site-specific associations: CHEK2 in high-grade ovarian cancer was associated with intra-abdominal (OR = 6.44), liver (OR = 12.43), lung (OR = 15.22), and lymph node metastases (OR = 9.06); TP53 in ovarian cancer predicted intra-abdominal spread (OR = 4.07); PPM1D and TET2 in cutaneous melanoma were linked to liver metastases (OR = 13.20 and OR = 6.39, respectively); ATM in bladder cancer predicted lung metastases (OR = 7.89), while PPM1D in bladder and lung squamous carcinoma was associated with lymph node involvement (OR = 3.55 and OR = 4.26, respectively). Parallel analyses of ctDNA mutations revealed tumor-intrinsic drivers of organotropism, including TP53 mutations in breast ductal carcinoma (bone metastases, OR = 39.95), KRAS in pancreatic adenocarcinoma (hepatic spread, OR = 7.50), and FOXA1 in prostate adenocarcinoma (liver involvement, OR = 45.39). Figure created with BioRender.com.

To explore whether somatic mutations in CHIP or ctDNA were associated not only with the presence of metastases but also with their anatomical distribution, we performed a tropism analysis across cancer types. Multinomial logistic regression models were applied separately to CHIP and ctDNA mutation data using the VGAM package in R, with metastatic site as the categorical outcome and “Primary” tumor location as the reference. Models included age and gender as covariates (see Methods). Analyses were limited to cancer types with ≥50 patients and at least three distinct metastatic sites (Fig. 5; Tables S7–S8, available as supplementary data at *Bioinformatics* online).

Among CHIP-derived mutations, several DNA damage response (DDR) and epigenetic regulator genes displayed striking associations with specific sites of metastasis. In high-grade ovarian cancer, CHEK2 mutations were strongly associated with increased odds of metastases to the intra-abdominal space (OR = 6.44, *P* = 0.027, *n* = 20), liver (OR = 12.43, *P* = 0.026), lungs (OR = 15.22, *P* = 0.033), and lymph nodes (OR = 9.06, *P* = 0.017), while TP53 mutations also predicted intra-abdominal spread (OR = 4.07, *P* = 0.031, *n* = 23), reinforcing the relevance of DDR gene dysfunction in systemic dissemination.

In cutaneous melanoma, PPM1D (OR = 13.20, *P* = 0.044, *n* = 10) and TET2 (OR = 6.39, *P* = 0.032, *n* = 21) mutations were significantly associated with liver metastases, suggesting a potential link between epigenetic dysregulation and non-canonical metastatic routes.

Several additional associations highlighted possible gene-specific tropisms: in bladder cancer, ATM mutations predicted lung metastases (OR = 7.89, *P* = 0.033, *n* = 8), and PPM1D mutations were associated with lymph node involvement (OR = 3.55, *P* = 0.028, *n* = 37). In lung squamous carcinoma, PPM1D again correlated with nodal spread (OR = 4.26, *P* = 0.023, *n* = 40).

Conversely, certain mutations were associated with reduced risk of metastasis to specific organs. In breast lobular carcinoma, PPM1D mutations were linked to lower odds of skin metastases (OR = 0.088, *P* = 0.043, *n* = 7), and in pancreatic adenocarcinoma, MITF mutations were associated with decreased hepatic (OR = 0.0077, *P* = 0.0079, *n* = 5) and soft tissue spread (OR = 0.0014, *P* = 0.0011). Finally, in prostate cancer, EPHA3 mutations were associated with reduced retroperitoneal dissemination (OR = 0.0027, *P* = 0.0037, *n* = 5).

Parallel analyses of ctDNA mutations revealed complementary tumor-intrinsic associations. In breast ductal carcinoma, TP53 mutations were significantly enriched in patients with bone metastases (OR = 39.95, *P* = 0.032, *n* = 57), in line with the known role of TP53 dysfunction in promoting invasive phenotypes. In lung adenocarcinoma, MAP2K1 mutations were inversely associated with soft tissue metastases (OR = 0.00016, *P* = 0.0037, *n* = 6), suggesting a potential protective effect or subtype-specific metastatic behavior. In pancreatic adenocarcinoma, KRAS mutations, present in a high fraction of patients were strongly associated with liver metastases (OR = 7.50, *P* = 0.0074, *n* = 106), reinforcing the central role of RAS signaling in hepatic seeding. Finally, in prostate adenocarcinoma, FOXA1 mutations conferred markedly increased odds of liver involvement (OR = 45.39, *P* = 0.0450, *n* = 9), implicating this lineage-specific transcription factor in the organotropism of advanced disease.

Taken together, these findings reveal that somatic mutations detectable in peripheral blood, whether arising from clonal hematopoiesis or circulating tumor DNA, carry distinct, non-redundant information about metastatic site preference (Fig. 5; Tables S7–S8, available as supplementary data at *Bioinformatics* online). CHIP mutations appear to reflect systemic, host-derived influences, while ctDNA captures tumor-intrinsic drivers of organ-specific dissemination. Their integration offers novel insight into the biology of metastasis and the systemic environment shaping its trajectory.

### 3.6 Variant-level characteristics of CHIP and ctDNA mutations reveal distinct biological patterns

To further dissect the biological nature of blood-derived mutations, we performed three orthogonal analyses that considered variant-level features beyond gene identity. We analyzed (i) positional hotspots, (ii) clonality as measured by variant allele frequency (VAF), and (iii) per-patient mutational burden. These analyses revealed important differences between CHIP and ctDNA and highlighted context-specific mutation dynamics.

First, we evaluated whether specific mutations occurred recurrently at identical genomic positions (hotspots), indicative of convergent selection (Table S9, available as supplementary data at *Bioinformatics* online). ctDNA mutations showed a clear enrichment at canonical oncogenic hotspots across multiple tumor types. Notably, EGFR alterations such as p. E746_A750del and p. L858R, KRAS mutations including p. G12C, p. G12D, and p. G12V, as well as TP53 codon substitutions (e.g. p. R273C, p. R248Q), were among the most frequently mutated positions. These findings are consistent with strong selective pressures for functional driver mutations within the tumor genome. In contrast, CHIP mutations, while still showing some recurrences, were more concentrated within a narrower set of genes, particularly DNMT3A, which accounted for the majority of the top hotspot events (e.g. p. R882H, p. R882C, and various splice or truncating variants). Additional recurrent sites included PPM1D truncating mutations (e.g. p. R552, p. R572) and frameshift alterations in ASXL1. This mutation pattern suggests that clonal hematopoiesis is driven by selective forces favoring hematopoietic clonal advantage, such as resistance to cellular stress or replicative benefits rather than classical tumorigenicity.

Second, we assessed the prognostic value of variant allele frequency (VAF) for CHIP and ctDNA mutations, modeling both the presence of mutations and their clonal size (Tables S10–S11, available as supplementary data at *Bioinformatics* online). Across multiple cancer types, higher VAFs of CHIP mutations, particularly in genes such as DNMT3A, TET2, and PPM1D were associated with worse overall survival in several cancer types, even after adjusting for the mere presence of the mutation. These findings support the hypothesis that expanded hematopoietic clones may exert systemic effects that influence cancer progression, potentially through inflammatory or immunomodulatory mechanisms. In contrast, while many ctDNA mutations also showed prognostic associations with VAF, these were frequently accompanied by a significant effect of mutation presence, suggesting that VAF in the ctDNA context often reflects underlying tumor burden or clonal dominance without necessarily providing additional prognostic information beyond mutation detection.

Third, we evaluated the prognostic impact of total mutational burden for CHIP and ctDNA, adjusting for age and gender size (Tables S12–S13, available as supplementary data at *Bioinformatics* online). CHIP mutational burden was significantly associated with worse overall survival in a subset of tumors, supporting the notion that the cumulative effect of expanded hematopoietic clones may influence disease progression. Similarly, ctDNA mutational burden emerged as a strong predictor of poor prognosis, likely reflecting underlying tumor burden and clonal complexity.

Together, these analyses extend our understanding of how mutation-level properties contribute to the prognostic value of circulating alterations. While ctDNA reflects tumor-intrinsic clonal evolution with recognizable driver hotspots and burden-associated risk, CHIP mutations demonstrate clonal expansion patterns with gene- and VAF-specific implications for outcome. These features may guide future biomarker development based on quantitative and positional characteristics of somatic variants.

### 3.7 Comparative analysis of CHIP-only, ctDNA-only, and joint models in lung adenocarcinoma

To more explicitly evaluate the potential complementarity between hematopoietic-derived and tumor-derived circulating alterations, we performed a side-by-side comparison of CHIP-only, ctDNA-only, and joint models in lung adenocarcinoma, the only tumor type with sufficient overlap between CHIP and ctDNA data to support interpretable integrative analyses. This analysis was intended as a proof-of-principle exploration of the dual-compartment framework rather than as a generalized pan-cancer predictive model. Consistent with the analyses presented above, CHIP-only models highlighted associations involving genes related to DNA damage response and epigenetic regulation, supporting a potential contribution of host-derived systemic processes to cancer progression and metastatic behavior. In contrast, ctDNA-only models identified tumor-intrinsic alterations associated with prognosis and metastatic dissemination, including mutations affecting canonical oncogenic signaling pathways.

When considered jointly, CHIP and ctDNA alterations appeared to provide complementary, potentially non-overlapping information regarding disease biology. In this context, CHIP-associated mutations may reflect systemic host-related processes, whereas ctDNA alterations directly capture tumor-intrinsic clonal evolution and disease burden. Together, these findings support the rationale for integrating both liquid biopsy compartments within a unified analytical framework.

Given the limited number of patients with fully matched CHIP, ctDNA, and clinical data across additional tumor types, we intentionally restricted this integrative analysis to lung adenocarcinoma to preserve statistical interpretability and avoid overgeneralization. Larger multi-compartment cohorts will be required to further evaluate the clinical utility of combined liquid biopsy models across diverse cancer contexts.

### 3.8 Clonal dynamics of CHIP in hematologic malignancies

**Figure 6:**
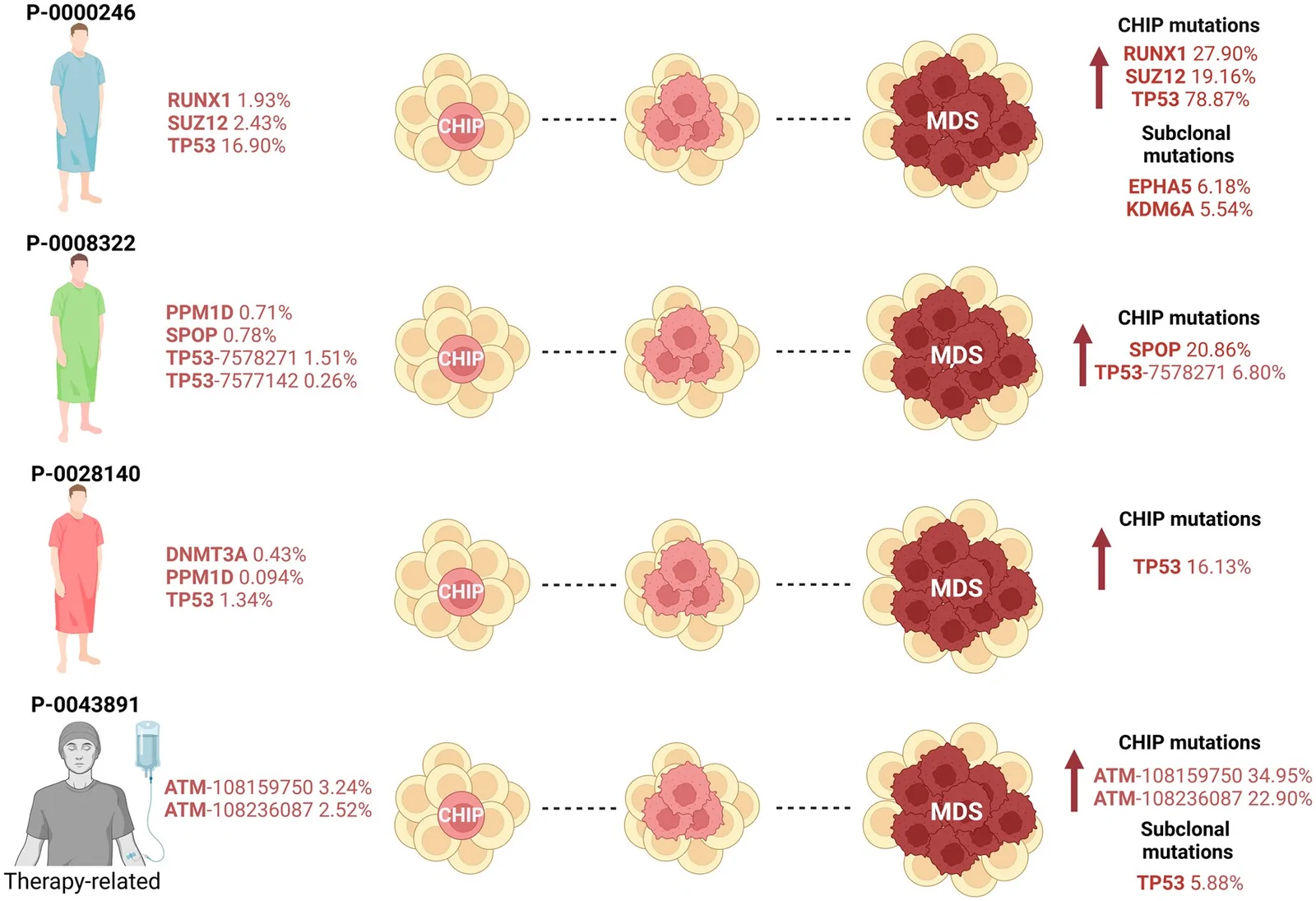
Clonal Evolution in Myelodysplastic Syndromes (MDS) from CHIP. This figure illustrates the clonal expansion trajectories for four selected MDS patients, including one case of therapy-related MDS (bottom). Each patient’s clonal dynamics are depicted, highlighting the progression from initial CHIP clones to the dominant clones present in MDS. This visualization underscores the role of CHIP in initiating hematologic malignancies and the impact of therapy on clonal selection. Figure created with BioRender.com.

Therapy-related myeloid malignancies represent a significant unmet clinical need in hematology, characterized by poor overall survival. It is hypothesized that some cases of therapy-related AML may arise from a pre-existing mutated clone, which, under selective pressure from chemotherapy or radiotherapy, expands and acquires additional driver mutations. However, tracking clonal evolution from CHIP to hematologic malignancies in real-world clinical contexts is challenging due to the lack of longitudinal datasets. Leveraging our cohort (Ptashkin *et al.* 2023, Stonestrom *et al.* 2024), we explored clonal dynamics by analyzing data from 32 patients with CHIP who later developed hematologic malignancies, of which 23 also presented a solid tumor diagnosis (Table S14, available as supplementary data at *Bioinformatics* online). The patterns of clonal expansion varied significantly between these cancer types. In 24 out of 26 myeloid malignancies, including 19 myelodysplastic syndromes (MDS), the tumors arose directly from an antecedent CHIP clone. This trend was particularly evident in one therapy-related MDS patient, where two distinct ATM mutations were observed in one CHIP clone, which later expanded under selective pressure from therapy (Fig. 6).

In contrast, lymphoid malignancies displayed a distinct pattern. CHIP mutations consistently disappeared during tumor progression in these cancers, with all cases arising from de novo mutations unrelated to the original CHIP clones.

In myeloid cancers, mutations in DDR genes such as TP53, ATM, and CHEK2 consistently expanded into the dominant tumor clone, reinforcing their role as early drivers of the malignancy. Notably, PPM1D mutations never expanded into the tumor clone, suggesting that while PPM1D mutations may drive early clonal hematopoiesis, they might not confer a selective advantage during tumor progression. However, given their known role in therapy resistance, these clones may still contribute to disease relapse.

Finally, we observed evidence of polyclonality in approximately 30% of patients, with multiple CHIP clones present. Yet, as hematologic malignancies progressed, most of these clones disappeared, with the malignancy arising from a single dominant CHIP clone that drove tumorigenesis.

## 4 Discussion

This study provides a large-scale, tumor-type–resolved assessment of ctDNA and CHIP in solid tumors, highlighting their complementary roles in reflecting tumor-intrinsic versus host-mediated biology. While our findings suggest that CHIP and ctDNA mutations convey distinct prognostic and metastatic information across cancer types, several limitations should be acknowledged. Survival and metastasis analyses did not adjust for tumor stage or ctDNA fraction, both strong predictors of outcome that may also influence ctDNA detectability, treatment exposure, and CHIP prevalence. Variant allele frequencies of CHIP and tumor mutations were also not corrected for tumor purity. These limitations largely reflect the availability and structure of clinical data in public cohorts, which were not uniformly curated for detailed covariates. Moreover, the MSKCC cohorts derive from a tertiary referral center and may therefore not fully represent the broader oncology population. In addition, given the exploratory and cross-tumor nature of the analyses, some associations involving genes mutated in relatively small patient subsets should be interpreted with caution and considered hypothesis-generating rather than definitive prognostic findings.

Furthermore, our study should be interpreted primarily as a large-scale association analysis rather than evidence of causal or fully stage-independent prognostic effects and does not directly address the mechanistic basis of how CHIP clones may contribute to organ-specific metastasis. Patient exposure to diverse therapies adds another layer of complexity, as treatment-related selective pressures may influence both ctDNA evolution and CHIP clonal expansion, potentially confounding observed associations with clinical outcomes. In addition, synonymous and non-coding variants were not included in the current targeted-panel-based analysis and may represent an important direction for future investigation. Repeated longitudinal ctDNA profiling may further help clarify whether detected alterations represent early tumor drivers or later therapy-associated evolutionary events. Future work integrating tumor stage, ctDNA fraction, treatment information, and mechanistic studies, together with variant-level analyses, will be essential to refine our understanding of dual-compartment dynamics and improve their translational potential in prognostic modeling, computational risk stratification, and therapy guidance.

## Supplementary Material

btag491_Supplementary_Data

## Data Availability

Public genomic and clinical data are available through cBioPortal; the analysis scripts and pipeline used to reproduce the results are available on GitHub: https://github.com/danro9685/Liquid-biopsies-study.
